# Assessing bird avoidance of high-contrast lights using a choice test approach: implications for reducing human-induced avian mortality

**DOI:** 10.7717/peerj.5404

**Published:** 2018-09-26

**Authors:** Benjamin Goller, Bradley F. Blackwell, Travis L. DeVault, Patrice E. Baumhardt, Esteban Fernández-Juricic

**Affiliations:** 1 Department of Biological Sciences, Purdue University, West Lafayette, IN, USA; 2 USDA/APHIS/WS National Wildlife Research Center, Sandusky, OH, USA

**Keywords:** Bird strikes, Visual modeling, Responses to lights, Bird collisions, Avoidance, Behavioral assay

## Abstract

**Background:**

Avian collisions with man-made objects and vehicles (e.g., buildings, cars, airplanes, power lines) have increased recently. Lights have been proposed to alert birds and minimize the chances of collisions, but it is challenging to choose lights that are tuned to the avian eye and can also lead to avoidance given the differences between human and avian vision. We propose a choice test to address this problem by first identifying wavelengths of light that would over-stimulate the retina using species-specific perceptual models and by then assessing the avoidance/attraction responses of brown-headed cowbirds to these lights during daytime using a behavioral assay.

**Methods:**

We used perceptual models to estimate wavelength-specific light emitting diode (LED) lights with high chromatic contrast. The behavioral assay consisted of an arena where the bird moved in a single direction and was forced to make a choice (right/left) using a single-choice design (one side with the light on, the other with the light off) under diurnal light conditions.

**Results:**

First, we identified lights with high saliency from the cowbird visual perspective: LED lights with peaks at 380 nm (ultraviolet), 470 nm (blue), 525 nm (green), 630 nm (red), and broad-spectrum (white) LED lights. Second, we found that cowbirds significantly avoided LED lights with peaks at 470 and 630 nm, but did not avoid or prefer LED lights with peaks at 380 and 525 nm or white lights.

**Discussion:**

The two lights avoided had the highest chromatic contrast but relatively lower levels of achromatic contrast. Our approach can optimize limited resources to narrow down wavelengths of light with high visual saliency for a target species leading to avoidance. These lights can be used as candidates for visual deterrents to reduce collisions with man-made objects and vehicles.

## Introduction

Birds from numerous species frequently collide with human structures and vehicles (e.g., buildings, cars, airplanes, power lines, etc.), resulting in a source of mortality that has been exacerbated by the increase in habitat loss/fragmentation and urban sprawl (Longcore & Smith, 2013; Loss, Will & Marra, 2015; Loss, 2016). Even environmentally-friendly sources of energy can be sources of avian mortality, as multiple species have been found to collide with wind turbines and solar panels (Smith & Dwyer, 2016). Lights have been proposed as beacons to alert birds and minimize the chances of collisions (Lustick, 1973; Larkin et al., 1975). Actually, different types of lights have been implemented in specific contexts with varying degrees of success (Conover, 2002; Blackwell, Bernhardt & Dolbeer, 2002; Blackwell & Fernández-Juricic, 2013). Although the idea is straightforward, it brings some conceptual challenges; many of which remain unresolved, hence limiting successful implementation.

Bird vision is different from human vision (Cuthill, 2006; Tanaka, 2015), and different bird species also differ in the way they perceive objects visually (Fernández-Juricic, 2016). The implication is that light characteristics (e.g., wavelength, pulsing frequency, viewing angle) that enhance the stimulation of the human retina may not necessarily stimulate the avian retina to the same degree. The lack of consideration of the differences in visual perception between humans and birds has led to choosing light characteristics for deterrents through a trial-and-error approach (reviewed in Blackwell & Fernández-Juricic, 2013), which can lengthen the time and increase the costs of finding successful solutions. The application of guiding principles from sensory biology to develop avian deterrents by learning about key visual properties of target species and then developing stimuli around those properties has gained more support recently (Lim, Sodhi & Endler, 2008; Blumstein & Fernández-Juricic, 2010). More specifically, establishing the visual “sweet-spots” of a species (e.g., areas of the visual space that enhance the stimulation of photoreceptors) and building lights that will over-stimulate these “sweet-spots” can optimize the use of limited resources (Fernández-Juricic, 2016).

Perceptual models are often used to understand how non-human species see their world (Tanaka, 2015). Multiple studies have measured key properties of the visual system of different bird species (Hart & Hunt, 2007) that can be used to parameterize perceptual models, which can predict the saliency of stimuli in relation to the visual background and ambient light conditions from the visual perspective of a given species (Vorobyev et al., 1998; Endler & Mielke, 2005). For example, perceptual models have successfully predicted the higher visual saliency of aircraft with lights on (relative to ones with the lights off) that led to a quicker alert response by Canada geese (Blackwell et al., 2012), one of the species with the highest frequency of damaging bird-aircraft collisions (hereafter, bird strikes; Dolbeer et al., 2016). Perceptual models can actually be used to define wavelength-specific “sweet-spots” for a particular species by establishing which lights have the highest values of visual contrast (i.e., high visual conspicuousness) relative to the visual background in the chromatic and/or achromatic visual dimensions.

However, high retinal stimulation is not necessarily associated with enhanced alert or avoidance behavior. Certain wavelengths of light could instead lead to an attraction response (Avery, Springer & Cassel, 1976; Gauthreaux & Belser, 2006; Rodríguez et al., 2015). If that were the case, the problem of collisions could be exacerbated rather than minimized (Blackwell et al., 2016). Therefore, it is imperative that the outcomes of perceptual modeling are complemented by behavioral experiments (Doppler et al., 2015). The *integration* of visual physiology, perceptual modeling, and standardized behavioral testing to measure responses to wavelength-specific lights has rarely been considered in the literature (but see Doppler et al., 2015 relative to lights, Raveh et al., 2012 relative to objects of different color).

Our purpose was twofold: establish wavelength-specific lights with a high probability of stimulating the retina through perceptual modeling, and test via a behavioral assay whether those high-contrast lights lead to avoidance, attraction, or neutral responses. We used brown-headed cowbirds (*Molothrus ater*) as the model species for several reasons: (a) they are commonly involved in bird strikes with aircraft (Dolbeer et al., 2016), (b) their visual systems have been characterized to a level in which we can develop species-specific perceptual models (Fernández-Juricic et al., 2013), and (c) they have been shown to respond via alert behavior to lights tuned to their visual system (Doppler et al., 2015).

Measuring avoidance/attraction is extrapolated from choice behavior tests (Fechner, 1860/1966; Green & Swets, 1966; Manly et al., 2002). A choice test requires exposing an animal simultaneously to different stimuli and recording which option is selected by following specific criteria (e.g., amount of time spent on one option over the other, direction of movement; Amdam & Hovland, 2011). Some observational studies attempted to measure bird avoidance of lights (Gehring, Kerlinger & Manville, 2009; Foss, Ronning & Merker, 2017; Rodríguez, Dann & Chiaradia, 2017). However, their study designs did not allow for the simultaneous exposure to alternative stimuli to a given bird, preventing determination of cause-effect relationships involved in choice behavior. Additionally, these studies did not appear to control (manipulatively or statistically) for variations in potentially confounding variables such as the spatial surroundings that could affect risk-taking behavior, ambient light conditions that could influence the perception of the lights, the identity of the animals that could lead to pseudoreplication, and the time animals had been exposed to the light treatments before making a decision that could modify their choices. Inferences about avoidance of lights without the proper methodology to measure choice behavior can be misleading, which could result in negative implications for policy, safety, and product development. For instance, if an agency decides on a particular light color to minimize bird strikes based on a single study without any kind of standardized choice test, companies could produce a product that might work in some environmental contexts but not in others, which could lead to wasteful spending, damage in reputation, and even higher frequency of bird collisions. Our study can be deemed as a first step in addressing this gap.

In a potential collision scenario, birds are expected to face an imminent threat forcing them to make rapid decisions to increase the chances of evading the object and ultimately surviving (Bernhardt et al., 2010), which would require rapid integration of the sensory input with motor control (Sun & Frost, 1998; Card & Dickinson, 2008; Eckmeier et al., 2008; De Vries & Clandinin, 2012). In such situations, rapid assessments based on salient sensory cues (e.g., lights) can be a driving factor in decision-making as animals would not have time to explore alternatives, as compared to foraging or mate-choice scenarios. We developed a behavioral assay to test quick decision-making under diurnal light conditions by releasing a bird into an experimental arena in a way that it moved in one direction until it was forced to make a choice of going right or left (with different visual cues associated with each side). Our main motivation was to identify lights that would lead to avoidance responses ultimately to minimize collisions with man-made objects and/or vehicles; consequently, we used a single-choice (also known as no-choice) design (Withers & Mansfield, 2005; Van Driesche & Murray, 2004) in which one side had a light turned on and the other side had a light turned off. Single-choice designs have been shown to perform better than two-choice (i.e., both sides with lights on) or four-choice (i.e., each option with lights on) designs to measure avoidance behavior (Lee & Forschler, 2016).

## Methods

### High-contrasting lights from the cowbird’s eye

To identify lights that would be highly conspicuous to cowbirds, we first ran perceptual models using light emitting diode (LED) light spectra. We developed 201 simulated individual LED spectra with peak wavelengths ranging from 300 to 700 nm (the extent of most birds’ visual range; Hart, 2001) with peaks at two-nm intervals. The spectra were based on actual data from a Super Bright LEDs Inc. (St. Louis, MO, USA) light with a 450-nm peak, which was measured with StellarNet Black Comet spectroradiometer from 300 to 700 nm with the integration time set to maximize the sensitivity and reduce saturation of the spectroradiometer. We then shifted the peak of the spectrum, while maintaining the shape of the spectrum curve, to the different wavelength and normalized the intensity of the spectra to high (60,000 relative photon counts), medium (30,000 relative photon counts), and low (5,000 relative photon counts) intensity levels to establish the variation in chromatic and achromatic contrasts relative to variations light intensity.

We measured the spectral properties of ambient light and radiance of the visual background in an open grassy field in West Lafayette, Indiana (40°25′02.9″N, 86°56′29.5″W) outside of the Purdue University Airport, as this was the location of a previous behavioral study assessing cowbird behavioral responses to LED lights mounted on a radio-controlled aircraft (Doppler et al., 2015). We collected ambient light data using the absolute irradiance module from an Ocean Optics Inc. (Largo, FL, USA) Jaz spectrometer from 300 to 700 nm on a sunny day (<10% cloud cover; March 21st, 2015) and on a cloudy day (>80% cloud cover; March 19th, 2015) in 1 h intervals from dawn (08:00 h) until dusk (20:00 h) EST. We chose these times because our behavioral assay was conducted during daylight. At each time point, we measured the absolute irradiance twice from the four cardinal directions and the sky directly above the observer, for a total of 10 measurements, which were averaged, converted from μW/cm^2^/nm to μmol/s/m^2^ (the units required in the contrast calculations), and interpolated in one-nm intervals. We measured the radiance of the sky (i.e., visual background) at 45° from the ground from 300 to 700 nm using the spectroscopy module on the Jaz spectrometer with a set integration time at 30 ms for all radiance measures. At each time point, we measured the radiance twice from each of the four cardinal directions and the sky directly above the observer, for a total of 10 measurements, which were averaged and interpolated at one-nm intervals.

To make the perceptual models cowbird-specific, we incorporated the sensitivity of the brown-headed cowbird visual system by using the photon capture probability (C_r_ (λ); Endler & Mielke, 2005) and cowbird relative densities for each photoreceptor type (UVS:SWS:MWS:LWS:Double Cone = 1:6.19:6.91:5.78:16.8) from Fernández-Juricic et al. (2013). Calculating the photon capture probability (C_r_ (λ)) requires the cone photoreceptor visual pigment absorbance spectrum (*G_r_*(λ)), the transmission spectrum of the cone’s oil droplet (*T_or_*(λ)), and the transmission spectrum of the ocular media (*T_e_*(λ)) (Eq. (8), Endler & Mielke, 2005). We calculated the cone photoreceptor visual pigment absorbance spectrum (*G_r_*(λ)) using Eqs. (1), (2), and (5b) from Govardovskii et al. (2000) using λ_max_ (nm) values from the following visual pigments; UVS = 369 nm, SWS = 475 nm, MWS = 506 nm, LWS = 573 nm (Fernández-Juricic et al., 2013). We calculated the cone oil droplet transmission spectrum (*T*_or_(λ)) by using Eq. (17) from Hart & Vorobyev (2005) and λ_cut_ (nm) and B_mid_ values, respectively, from the following oil droplets; C-Type = 418 nm and 0.034, Y-Type = 516 nm and 0.026, R-Type = 576 nm and 0.033, P1-Type = 436 nm and 0.027 (Fernández-Juricic et al., 2013). We measured the average cowbird-specific transmittance spectrum of the ocular media (*T_e_*(λ); λ_T0.5_ = 312 ± 0.58 nm following Hawryshyn, Chou & Beauchamp, 1985; Hart et al., 2000; Hart, 2004) from three eyes (one left, two right) of two individuals (one male, one female). We used Vorobyev and Osorio’s tetrachromatic perceptual model (Vorobyev & Osorio, 1998) in Pavo v0.5–1 (Maia et al., 2013) to estimate both the chromatic and achromatic contrast (in Just Noticeable Differences, JNDs) of each LED light at three intensity levels against a sky background on sunny and cloudy days in 1 h intervals. For these calculations, we set the Weber fraction to 0.1 (following Vorobyev et al., 1998; Lind, Chavez & Kelber, 2014).

**Figure 1:**
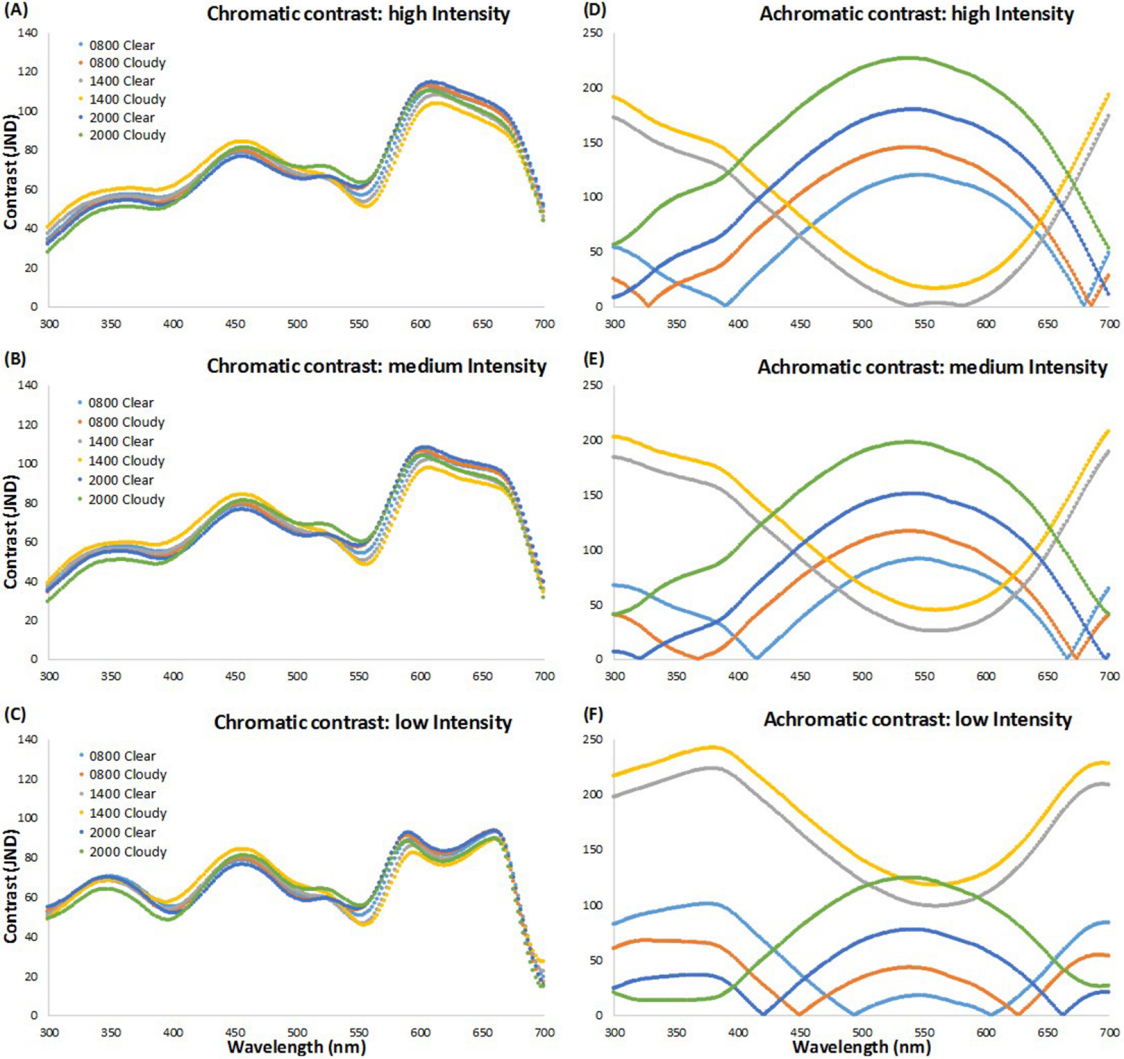
Chromatic and achromatic contrast of simulated LED lights. Scatterplot of chromatic and achromatic contrast of simulated LED lights presented every two nm (see text for details). Chromatic (A–C) and achromatic (D–F) contrast values of each simulated LED from 300–700 nm at three LED light intensity levels (high (A, D), medium (B, E), and low (C, F)) against a clear and cloudy sky at dawn (08:00 h), midday (14:00 h), and dusk (20:00 h).

Figure 1 shows the chromatic and achromatic contrast values of each LED light from the visual perspective of brown-headed cowbirds at the three light intensity levels used (high, medium, low) against a clear and cloudy sky at dawn (08:00 h), midday (14:00 h), and dusk (20:00 h). The results for the other day times we recorded are similar to the midday values and are not presented here for ease of viewing. We found that the highest chromatically contrasting LEDs, considering different LED intensity levels and times of the day, ranged from 430 to 490 nm and 570 to 690 nm (Figs. 1A–1C). We also found that the highest achromatically contrasting simulated LEDs ranged approximately from 405 to 650 nm at high intensity, 403 to 630 nm at medium intensity, and 300 to 430 nm and 655 to 700 nm at low intensity at dawn (08:00 h) and dusk (20:00 h), and from 300 to 480 nm and 630 to 700 nm at all other time points and LED intensity levels (Figs. 1D–1F). Based on these results from our simulated LEDs, we decided to use LEDs that would have relatively high levels of both chromatic and achromatic contrast in different portions of the spectrum (i.e., >10 JNDs, with the detectability threshold being JND = 4, Siddiqi et al., 2004, and assuming that higher JND values would be associated with enhanced behavioral responses; Fleishman et al., 2016). We also incorporated white LEDs (i.e., lights with a broad spectrum in different portions of the visible spectrum) as these lights are widespread in commercial aircraft applications.

**Figure 2:**
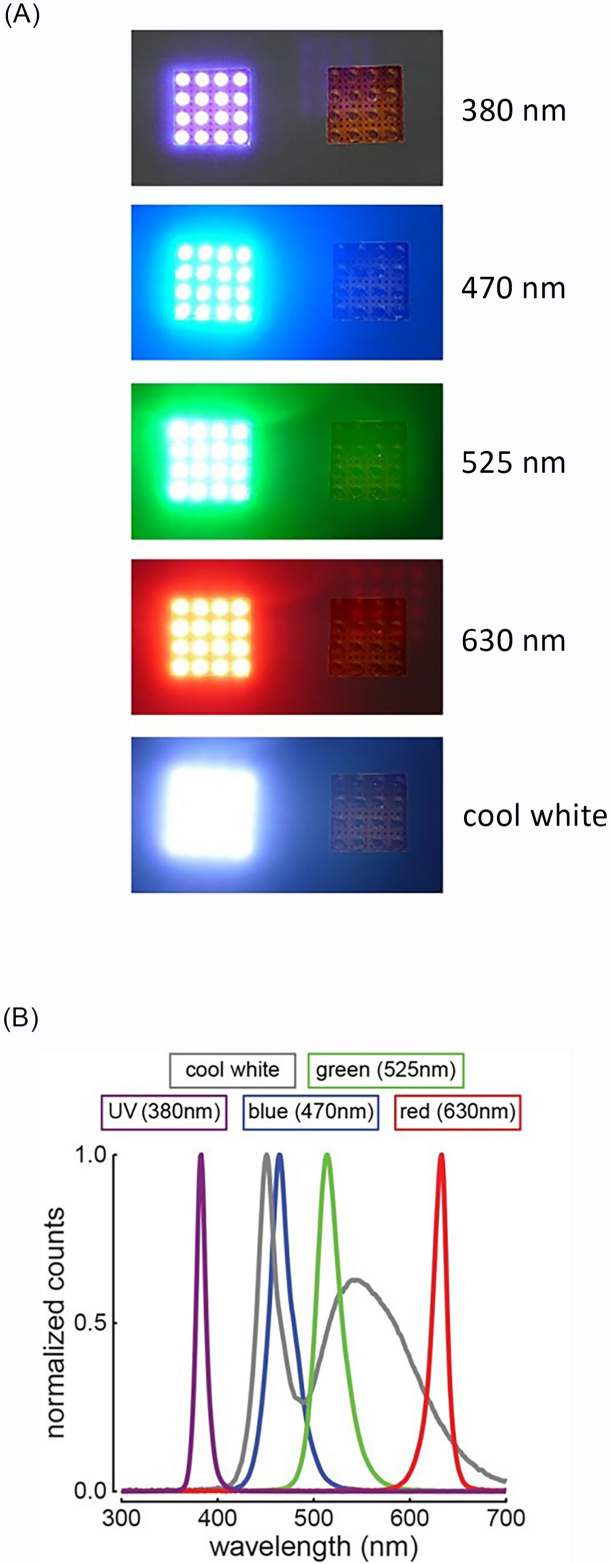
Light treatments. LED light treatments. (A) Photos of the five light-on conditions (380, 470, 525, 630 nm, and cool white) shown along with the paired light-off conditions (pictures taken by the authors). (B) Spectrophotometric measurements of the light emitted from each LED light type normalized for comparisons.

We were limited by the availability of LEDs in the market that would provide information on the spectral properties of the products as well as their viewing angles. We found lights that approached our specified conditions at Super Bright LEDs Inc. (St. Louis, MO, USA): five mm through-hole format LEDs with peaks at different wavelengths: 380-nm ultraviolet (RL5-UV0230-380), 470-nm blue (RL5-B2430), 525-nm green (RL5-G7532), 630-nm red (RL5-R8030), and “cool” white (RL5-W18030). The viewing angles for these five LEDs were 30°, 30°, 32°, 30°, and 30°, respectively. We were unable to purchase LEDs at these wavelengths with the exact same viewing angle; however, we did not consider the slight variation in viewing angle meaningful enough to bias our results. All LEDs looked identical to us in the light-off condition (Fig. 2A).

**Figure 3:**
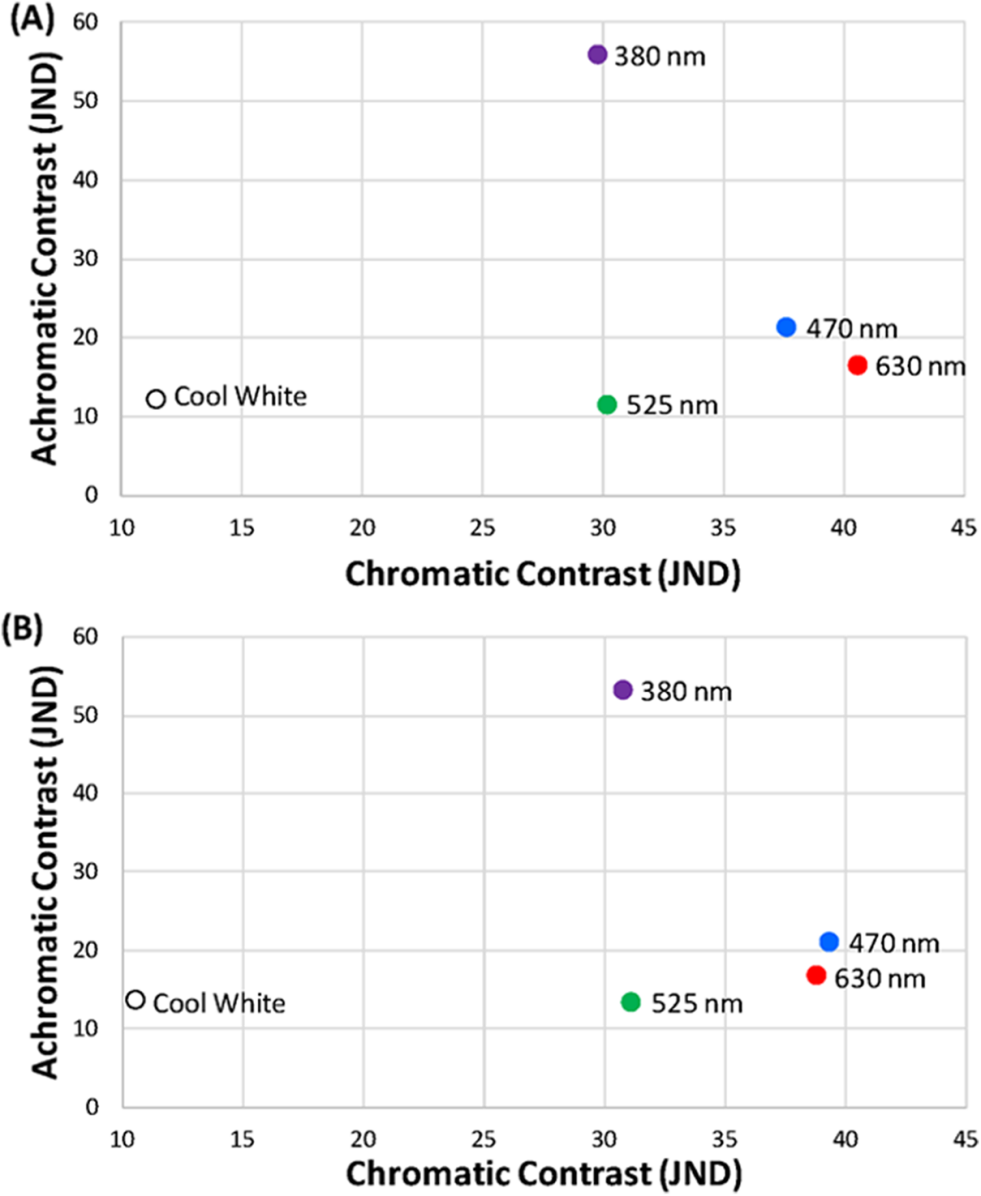
Light treatments in visual space. Position of treatment LED lights (380 nm ultraviolet, 470 nm blue, 525 nm green, 630 nm red, and cool white) in the achromatic and chromatic contrast space on (A) sunny and (B) cloudy ambient light conditions.

We measured the spectra of purchased LEDs using a StellarNet Black Comet spectroradiometer at a distance of 2.9 m from the surface of the LED using the average of ten 30 ms sweeps. The 2.9 m distance was required to prevent the brightest LED panel (UV) from saturating the probe. Thus, all LEDs were then measured at the same distance. We took 20 radiance measurements of each LED from 300 to 700 nm and averaged them for use in the contrast calculations. We confirmed that the LEDs emitted light spectra was close to the specifications of the manufacturer (Fig. 2B). The 380-nm UV LED peaked at 383 nm, the 470-nm blue LED peaked at 464 nm, the 525-nm green LED peaked at 514 nm, and the 630-nm red LED peaked at 633 nm. The width of the curves for each LED at 50% of peak emittance was measured as 377–388 nm (width = 11 nm), 453–478 nm (25 nm), 501–529 nm (28 nm), and 623–639 nm (16 nm) for UV, blue, green, and red LEDs, respectively (Fig. 2B). For the cool white LED, the peak emittance was 451 nm (width = 151 nm; 440–467 and 513–591 nm), but this cannot be directly compared to the other four because of the complex bimodal shape of the white LED emittance curve (Fig. 2B). We performed the same type of contrast calculations as described above but with the specific LED lights chosen for this study as the object of interest and the sky as the background under both sunny and cloudy ambient light conditions (Fig. 3). We confirmed that our LED lights used for the experiment met the aforementioned high-contrasting conditions in both the achromatic and chromatic dimensions.

### Behavioral assay to measure avoidance/preference

We conducted our behavioral experiment during daylight conditions using 33 wild-caught adult cowbirds (30 males, three females) that were housed in outdoor aviaries at the Ross Biological Reserve in Tippecanoe County, Indiana (40°24′35.16″N, 87°4′9.71″W). Individual cowbirds were identified by colored plastic leg bands. Cowbirds were given ad libitum water and food (millet, sunflower seed, bird chow, and mealworm mix). Animals were not food deprived during the days they were exposed to the treatment conditions. All experimental procedures were approved by the Institutional Animal Care and Use Committee at Purdue University (PACUC# 1401001019).

**Figure 4:**
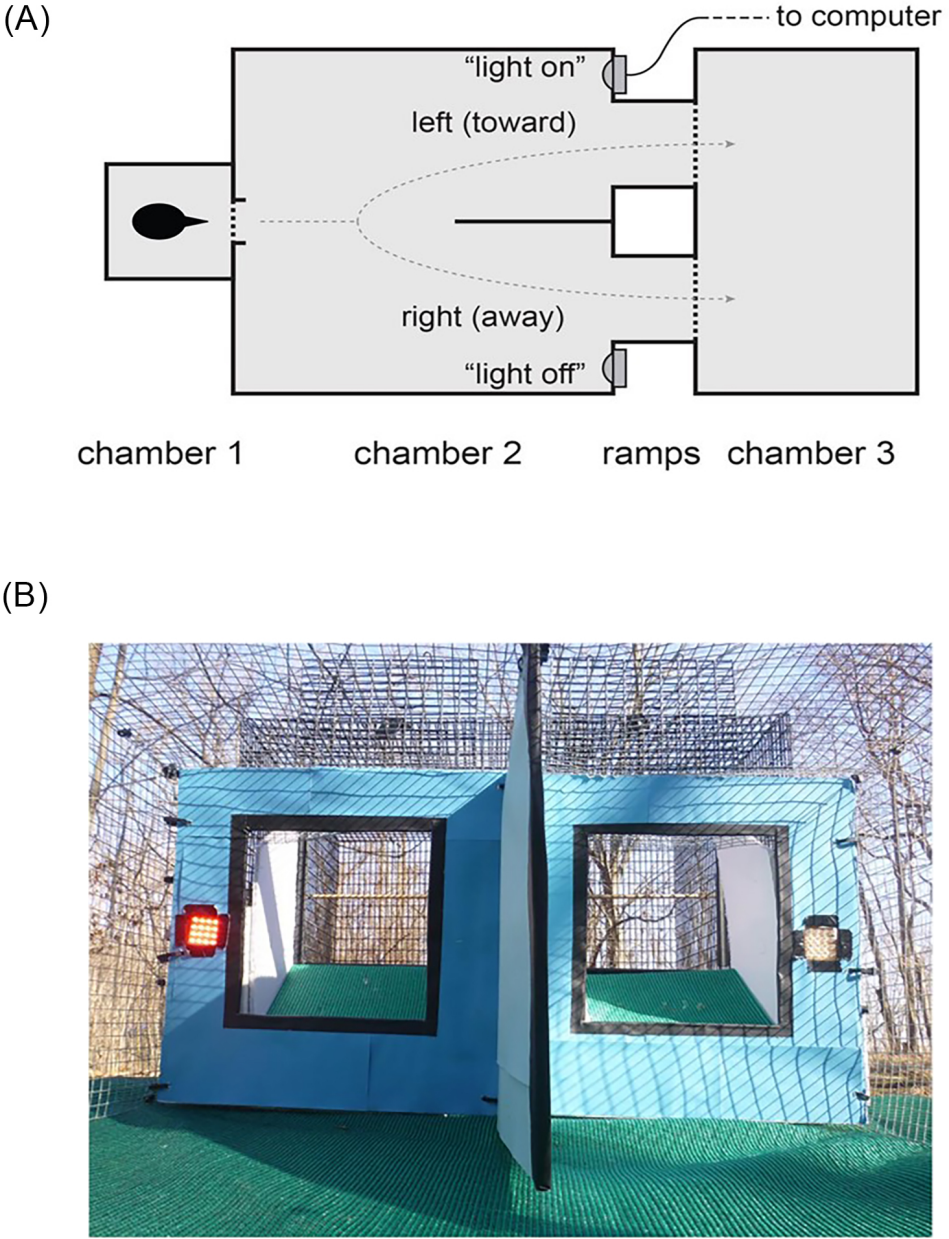
Experimental arena. (A) Cowbirds were released into chamber 1, and they then entered chamber 2, where they could choose a left or right exit to reach chamber 3. The two sides of chamber 2 were separated by a divider. (B) Photo from the entry into chamber 2 showing an example of one of the treatments: the light-on (630 nm LED) condition on the left side and the light-off condition on the right side (picture taken by the authors).

The experimental arena was placed in a small forest clearing at the Ross Biological Reserve and consisted of three chambers (Fig. 4A). In the first chamber (0.23 × 0.23 × 0.24 m, 13 × 25 mm wire mesh), the birds were held for less than 1 min. The back wall, sides, and top of this first chamber were covered with paper to remove external visual cues. The front was uncovered and had a door that opened into a second chamber (0.77 × 0.77 × 0.38 m) made of 1.27 cm hardware cloth (Figs. 4A and 4B). The far side of this second chamber was covered with blue paper to create a uniform background and split with a 0.30 m long divider (Fig. 4B). To the left and right of the divider, there were LED lights and 0.20 × 0.20 m windows (Fig. 4B). The windows led to two 0.16 m long ramps at 30° angles (Fig. 4B) that allowed the individual to fly into a third chamber (0.62 × 0.62 × 0.77 m, made of 13 × 25 mm wire mesh, Fig. 4A). Both the left and right side ramps led to the same endpoint (Fig. 4A). A single camera (GZ-E10BU camcorder, JVC) was placed above the arena to record the behavior of the individual.

Subject cowbirds were individually caught from the home aviaries and placed in loose cloth bags for 2–3 min before the behavioral trial. The bird was removed from the bag and placed into the first chamber of the experimental arena (Fig. 4). The observer was positioned behind the first chamber and started video recording and checked the stimulus lights from this position. Once the recording was started, the observer waited 5 s, then opened the door on the first chamber using a rope and the bird flew into the second chamber (Fig. 4). In the rare event that the bird did not exit the start cage, the observer tapped on the paper covered wall of the first chamber to make rattling sounds and/or repeatedly raised and lowered the start cage door with the attached rope. In most cases, subject cowbirds proceeded through the choice test. We did not consider data in which the animals did not enter the choice arena. After the choice test, we retrieved the individual from the third chamber and returned it to the home aviary.

To establish whether birds showed any preference for a given arena orientation (right, left) and whether that preference changed with compass direction without the light stimulus on, we first recorded choices for the right or left arena when lights were off on both the right and left sides (with the arena facing either east or west). We did this in two phases. In the first phase, seven cowbirds were tested twice each in each compass direction such that each individual was involved in four trials. In the second phase, we used 11 different cowbirds, but this time each individual was involved in a single trial in which the orientation of the arena was either east or west (randomly assigned). To combine data from these two phases and make them comparable, we randomly sampled one of the four trials for each of the seven first-phase individuals 1,000 times. The result was 1,000 groups of 18 trials where each of 18 cowbirds (pooling phase 1 and 2) had one choice of left or right side of the arena.

For the light attraction/avoidance experiment, the arena was set up randomly facing either east (10 trials) or west (10 trials) for a given light stimulus (see below for details). In addition, for a given compass direction (10 trials), the light-on stimulus was randomly placed on either the left (five trials) or the right (five trials) side of the chamber. For each trial, the stimulus light (light-on, either right or left) was always paired with a light-off (either left or right).

We tested five different light wavelengths and an individual cowbird was exposed to each wavelength only once. We randomly assigned the compass direction of the arena (east or west), and the left-right orientation of the light-on and light-off conditions, such that each of the four light-on orientation (right, left) and arena compass direction (east, west) combinations was repeated five times with five different birds. For instance, five cowbirds were exposed to a 630-nm LED light on the left when the arena faced east, five different cowbirds were tested with a 630-nm LED light on the right when the arena faced east, five with a 630-nm LED light on the left and arena facing west, and finally five cowbirds were tested with a 630-nm LED light on the right with the arena facing west. In total, we conducted 20 trials with 20 different cowbirds for each of the five LED panels. We chose a sample size of 20 individuals because a power analysis using pwr.p.test in R (Champely, 2016) showed that 20 individuals would yield a power of 80% for a significant (*P* = 0.05) result if 75% of the birds avoided/preferred the light-on treatment.

To produce a stimulus panel of a given peak wavelength, we arranged 16 LED bulbs of a single wavelength type in a 4 × 4 array (3 cm by 3 cm; Fig. 2A). Each panel was powered and controlled by an Arduino UNO R3 development board (www.arduino.cc) and two 74HC595D-T shift registers (Nexperia, Nijmegen, Netherlands) that were connected through USB to a laptop. The Arduino board provided a 5 V output and the LED bulbs were paired with resistors to maximize brightness (and therefore contrast) of each LED panel. To that end, we used the following limiting resistors: 11 Ω resistor for the UV LED, 21 Ω resistor for the blue and green LEDs, 51 Ω resistor for the red LED, and 15 Ω resistor for the white LED. The LEDs flickered at 10 kHz, but this rate was deemed not detectable by birds, as the highest reported flicker fusion frequencies are several orders of magnitude below the level used (Bostrom et al., 2016). Therefore, cowbirds were expected to see our treatments as steady LED lights.

We collected data on temperature and relative humidity from hourly measurements recorded at the nearby Purdue Airport weather station. We took light level measurements onsite by placing the probe of a TekPower LX1330B light meter (Kaito Electronics, Inc., Montclair, CA, USA) on the top of the second chamber above the left and right sides on three separate days for a total of eight measurements on the left side, and eight measurements on the right side of the arena. The range of light level measurements was 4600–110000 Lux. The forest canopy created a patchy mix of sunlight and shaded areas within the arena. The left and right sides of the arena were therefore scored as either “shaded” or “sunlit” by visual inspection of the videos to determine whether the left and right ramps leading to third chamber were covered by at least 50% shade or sunlight, respectively. Sides of the arena independently coded as “shaded” corresponded with mean light level measurements of 10,530 Lux, whereas those coded as “sunlit” corresponded with 52,583 Lux and were significantly brighter than the “shaded” sides (Welch’s unequal variances *t*-test: *t* = −3.01, d*f* = 6.031, *P* = 0.0236; R Core Team, 2016). Experiments were ended for the day in case of rain, but at least two individual trials were finished with raindrops starting to fall.

During the experiment, all behavioral responses were recorded as either left choice or right choice. In addition, we labeled the choices as toward or away from the light-on treatment, as well as north–south (north would be a left choice for the east arena orientation and right for the west arena orientation). The three ways of describing each choice corresponded to three alternative ways of categorizing the orientation of the ramps connecting chambers 2 and 3: toward-away referred the light-on vs. light-off treatment, left-right was a focal bird-centered categorization, and north–south referred to the vegetation structure configuration around the arena. This categorization of the data allowed us to establish the potential effects of not only the treatment of interest but also potential biases relative to the spatial orientation of the arena on cowbird attraction/avoidance behavior. Choice descriptions were verified by watching the videos of each trial.

In some cases, cowbirds immediately approached one ramp or the other, but did not fully cross into the third chamber. We counted these trials as if the individual had made a choice because the bird had crossed the divider that prevented them from seeing the other side of the cage. In the event that the subject flew back and forth, did not proceed past the divider, or spent its time trying to escape from the second chamber, the trial was abandoned and “no choice” was recorded (these data were not analyzed).

### Statistical analysis

To determine whether environmental factors affected cowbird choice behavior, we used a generalized linear mixed model (Knudson, 2017) with left and right choice as the binary dependent factor, and temperature, relative humidity, and shade condition as fixed factors, and individual bird as a random effect. We did not find evidence that these environmental factors significantly influenced left-right choice behavior in our study (temperature, *F*_1, 54_ = 1.34, *P* = 0.252; humidity, *F*_1, 54_ = 2.72, *P* = 0.105; shade *F*_3, 25_ = 0.58, *P* = 0.635), so we did not consider them further in our analyses.

To test whether cowbirds exhibited significant differences in their choices for different LED wavelengths, we used binomial tests (R Core Team, 2016) to determine whether cowbird choice was different from random (50% left and 50% right). To test whether the combination of compass direction of the arena and the left–right orientation of the light affected cowbird choice behavior, we used a Cochran–Mantel–Haenszel Chi-squared test (R Core Team, 2016) with east–west and left–right as independent factors.

## Results

In establishing the baseline response of cowbirds (light-off on both sides), we found that overall, cowbirds did not exhibit a preference for, or avoidance of, either the left or right sides of the experimental arena (in 21 out of 39 instances, individuals chose left, in 18 out of 39 instances, individuals chose right). The median result for the 1,000 simulated light-off data sets was 10 left choices (min = 7, max = 12) and eight right choices and we found no significant evidence of a preference or avoidance of either side (*P* median = 0.407, min = 0.119, max = 0.881).

**Figure 5:**
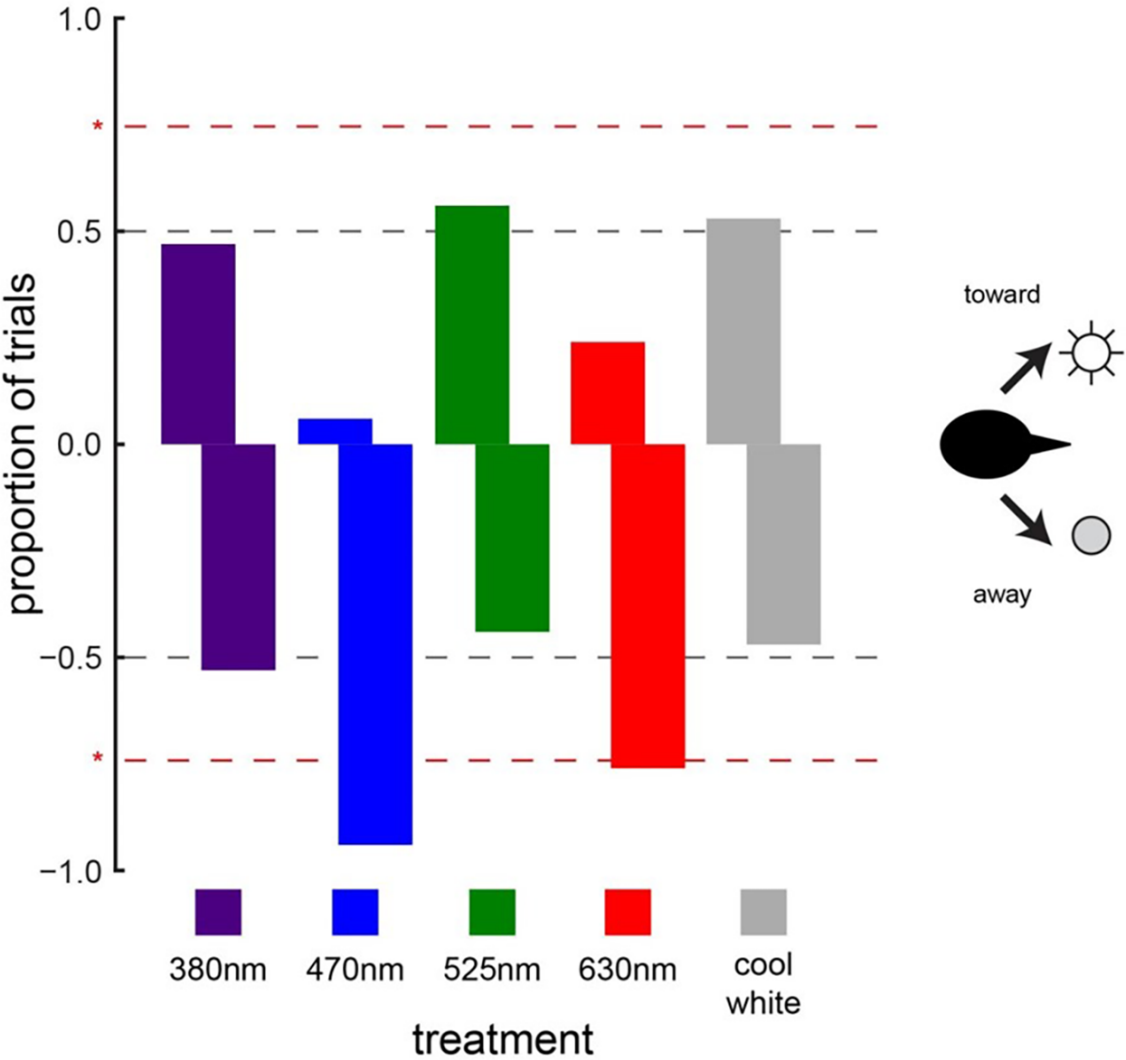
Cowbird responses to LED lights. Brown-headed cowbird responses to different LED lights. Red dashed lines indicate significant differences from random choice (50–50, which is illustrated with gray dashed lines). Positive values along the *y*-axis indicate attraction, whereas negative values indicate avoidance. Cowbirds showed significant avoidance of blue (470 nm) and red (630 nm) LED lights. Responses to ultraviolet (380 nm), green (525 nm), and white LED lights were random.

When animals were exposed to the light treatments (light-on vs. light-off) under daylight conditions, cowbird choice behavior was significantly affected by two of the five LED treatments tested (Fig. 3). Cowbirds significantly avoided the sides illuminated by the 470-nm LED (16/17, *P* = 0.000137) and the 630-nm LED (13/17, *P* = 0.0245) (Fig. 5). Cowbirds did not exhibit significant preference or avoidance for the 380-nm LED (9/17 avoided side with light-on, *P* = 0.500), the 525-nm LED (7/16, *P* = 0.773), or the “white” LED (8/17, *P* = 0.685) lights (Fig. 5).

We tested the role of other factors in the light treatment experiment. We found no significant difference between the number of left and right choices (64/123 chose left, *P* = 0.719). Instead of describing the choice as left–right, we also tested whether the birds preferred the choice to the north (left for east arena, right for west arena) or to the south, because of potential differences in the configuration of the vegetation structure around the arena, but found no significant differences (67/123 chose North, *P* = 0.367).

We also considered the potential simultaneous effects of light-on position (left–right) and arena orientation (east–west) on the responses of cowbirds, independent of the wavelength of the LED light used. Left choices (i.e., light avoidance) were significantly prevalent (16 left choices of 20 total trials) when the arena was facing east and when the light-on treatment was on the right side (Cochran–Mantel–Haenszel Chi-squared test, *Χ*^2^ = 4.957, d*f* = 1, *P* = 0.0260). However, this light avoidance effect in a particular arena orientation did not drive the wavelength-specific results. Specifically, removing the trials for the east facing arena with light-on treatment on the right for all wavelengths still yielded avoidance of 470 nm (12/13, *P* = 0.00171) and 630 nm (10/13, *P* = 0.0461), with no behavior pattern for 380 nm (6/13, *P* = 0.709), 525 nm (4/12, *P* = 0.927), and white lights (5/13, *P* = 0.867).

## Discussion

Our study integrates, for the first time, species-specific sensory modeling (based on previously published and new physiological data for brown-headed cowbirds) with a choice behavioral assay during daylight conditions to establish the wavelengths of highly visually contrasting LED lights that trigger avoidance behavior. Our assay simulates a quick decision scenario while the animal is moving in a particular direction, which might resemble situations in which birds encounter objects illuminated to different degrees (e.g., power lines, wind turbines buildings, communication towers, vehicles). However, we cannot generalize our results to all these human structures or vehicles because in our behavioral assay birds were presented with lights at very close distances (i.e., lights covered a large part of their visual field) and the responses may have changed with lights at farther distances. Nevertheless, our findings suggest that brown-headed cowbirds significantly avoid LED lights with peaks at 470 and 630 nm, but do not necessarily avoid nor prefer LED lights with peaks at 380 and 525 nm or broad-spectrum (white) LED lights.

An alternative way of characterizing choice behavior relative to lights is to run assays with lights peaking at every single wavelength of the avian visible spectrum. However, this could be logistically and financially challenging. Our methodological approach was designed to optimize resources by first narrowing down the wavelengths with high visual contrast (considering both chromatic and achromatic dimensions) using species-specific perceptual modeling and then assessing choice behavior of those portions of the spectrum (although we were limited in our choice by the availability of commercial LEDs). We propose that this approach could be used for other species in which there is a need to minimize collisions with human objects through the use of lighting systems, as it defines the lighting spectra necessary to elicit avoidance behavior.

We found that not all highly contrasting lights generated the same type of behavioral response. Cowbirds responded randomly (neither avoiding nor preferring) to ultraviolet (380 nm), green (525 nm), and white lights. This is particularly interesting because previous studies have suggested the use of these lights to reduce bird collisions. For instance, ultraviolet lights have been recommended to minimize collisions with wind turbines (May et al., 2017); green lights, with offshore platforms (Poot et al., 2008); and white lights, with communication towers (Gehring, Kerlinger & Manville, 2009). However, all these studies were observational in nature (i.e., recording wild bird behavior/mortality around objects with different types of lighting) and did not control for many of the factors that could lead to misleading results (i.e., variations in individual identity, hunger levels across individuals, flying heights across trials, etc.), nor did they provide animals with light choices that were spatially and temporally correlated. Without meeting these requirements (Foss, Ronning & Merker, 2017), it is challenging to make any conclusion about avoidance behavior (reviewed in Lee & Forschler, 2016). Our approach is a first step in minimizing these confounding effects, and reveals that previous conclusions about ultraviolet, green, and white lights as potential avian deterrents might need to be reexamined.

When cowbirds were exposed to LED lights with peaks at 470 nm (blue) and 630 nm (red), they chose to fly away from these lights. This is an intriguing finding because blue and red are on different ends of the wavelength spectrum tested. However, our cowbird-specific perceptual models revealed that these two LED lights happened to be the ones with the highest chromatic contrast of the ones considered (both >35 JNDs) and relatively similar levels of achromatic contrast (Fig. 3). Additionally, these two LED lights had much lower levels of achromatic contrast than the 380-nm (ultraviolet) lights used (Fig. 3). From the cowbird visual perspective, the implication is that the 470 nm (blue) and 630 nm (red) LED lights not only stand out from the visual background to a similar degree in both the chromatic and achromatic dimensions (Fig. 3), but also led to similar avoidance responses. Perceptual models like the one used in this study (Vorobyev & Osorio, 1998) were originally developed for establishing only visual detection thresholds (Kemp et al., 2015), but our results along with those of a recent study (Fleishman et al., 2016) suggest that they might work for predicting some behavioral responses relative to the strength of the sensory stimulation, at least in some very specific contexts. Obviously, much more fundamental research is necessary to test the association between suprathreshold stimuli and behavior (Kelber & Osorio, 2010; Kemp et al., 2015), but this work can better inform the development of novel stimuli to prevent bird collisions with objects.

Brown-headed cowbirds have been the subject of considerable research on their visual system and visual behavior (Blackwell & Bernhardt, 2004; Blackwell et al., 2009; Dolan & Fernández-Juricic, 2010; Fernández-Juricic et al., 2013; DeVault et al., 2015; Doppler et al., 2015; Ronald et al., 2017). Part of this work allowed us to develop species-specific perceptual models and narrow down the wavelengths with high chances of retinal stimulation. Further, one of the LED lights (470 nm) that caused avoidance responses in the present study was also found to shorten the time for cowbirds to detect a static object with lights-on vs. the same object with the lights-off (Doppler et al., 2015). Additionally, this 470 nm LED light reduced the effects of high speed of a radio-controlled aircraft on cowbird alert behavior (i.e., individuals exposed to vehicles at higher speeds showed alert behavior to an approaching aircraft with lights off later than compared to one with steady lights on; Doppler et al., 2015). Overall, LED lights peaking at 470 nm could be a good candidate to enhance the chances of cowbirds evading man-made objects, thereby reducing collisions, at least under daylight conditions. Future work should explore whether this avoidance is enhanced or maintained with LED lights at wavelengths around 470 nm and by changing features of the LED lights (e.g., pulsing rates and intensity). Measuring responses to different light stimuli can help to build a visual sensitivity map that relates different parameters (wavelength, luminance, pulsing rates, etc.) to behavioral performance (i.e., probability of avoiding lights of different wavelengths). Additionally, future work should assess how these visual sensitivity maps vary with repeated exposures over time (i.e., habituation/sensitization) and different weather conditions (variations in wind speed, precipitation, light intensity, etc.).

Our simulation and empirical studies reflected the diurnal activity patterns of Brown-headed cowbirds by considering only daylight conditions. However, the fact that some wavelengths generated avoidance responses during the day suggests that they might also work during the night. This could be particularly useful for songbirds migrating during the night (Van Doren et al., 2017) or for resident species whose behavior is altered by light pollution (Raap et al., 2017). Although we cannot generalize our results to these species and specific context, our approach can be adjusted in the future to assess this possibility by modeling the perception of the lights under night conditions and running behavioral assays in the dark.

Our study focused on lights, but it raises the interesting possibility that birds might also show avoidance behavior to certain wavelengths in bright painted surfaces. For instance, a correlational study using public records of bird-aircraft collisions found a negative association between brighter aircraft fuselage color and bird strikes (Fernández-Juricic et al., 2011). The use of painted surfaces would make the implementation of avian deterrents much easier; however, we caution that such possibility requires further empirical examination.

## Conclusions

If lighting is employed as a means of minimizing bird collisions with human-made structures and vehicles through enhanced detection and avoidance, we must understand how the visual conspicuousness of lights affects choice behavior. This is important because of the variation in the type and degree of behavioral responses of birds to different wavelengths of light (Gehring, Kerlinger & Manville, 2009; De Jong et al., 2015; May et al., 2017; Hunt, McClure & Allison, 2015). Our two-tiered methodological approach provides one way of tackling this complex problem, as behavioral responses to different suprathreshold visual signals can be highly variable. Our findings are limited only to steady lights under diurnal ambient light conditions and a single avian species. However, avian collisions occur in a wider range of conditions. For instance, species migrating at night face other challenges because the visual perception of the lights can be quite different and lights can actually act as attractants for navigational purposes (Poot et al., 2008; Van Doren et al., 2017). Additionally, different bird species can perceive lights very differently depending on their visual system configuration (Fernández-Juricic, 2016). We believe that our approach has the potential (with the proper adjustments) to be applied to this wide set of conditions and species.

## Additional Information and Declarations

### Data Availability

The following information was supplied regarding data availability: The raw data are included in the Figures.
